# Responses of dioecious *Populus* to heavy metals: a meta-analysis

**DOI:** 10.48130/FR-2023-0025

**Published:** 2023-10-24

**Authors:** Lei Yu, Shuanglei Tang, Jieyu Kang, Helena Korpelainen, Chunyang Li

**Affiliations:** 1 Department of Ecology, College of Life and Environmental Sciences, Hangzhou Normal University, Hangzhou 311121, China; 2 Department of Agricultural Sciences, Viikki Plant Science Centre, University of Helsinki, PO Box 27, FI-00014, Finland; 3 College of Agriculture and Biotechnology, Zhejiang University, Hangzhou 310058, China

**Keywords:** Heavy metals, Poplars, Sexual dimorphism, Reactive oxygen species

## Abstract

A total of 946 sets of comparative data were collected from 20 publications and a meta-analysis performed to evaluate the responses of growth, photosynthetic capacity, oxidative stress and antioxidants in *Populus* females and males under exposure to heavy metals, like Cu, Mn, Zn, Pb and Cd. It was found that heavy metals have negative effects on *Populus* growth and photosynthetic capacity, as the average total biomass, leaf biomass, stem biomass, root biomass and height decreased by 29.78%, 33.41%, 27.22%, 35.30% and 34.83%, respectively. Furthermore, total chl, *P*_n_, *g*_s_, *E*, *C*_i_ decreased by 23.30%, 26.03%, 40.49%, 23.76% and 18.24%, respectively. In addition, heavy metals increased oxidative stress and antioxidant enzyme activities: the average values of TBARS, H_2_O_2_, ${\text{O}^-_2} $ and MDA increased by 51.39%, 55.79%, 64.67% and 48.92%, respectively, and proline, APX, NPT, POD, CAT and SOD increased by 68.91%, 64.81%, 68.40%, 57.34%, 77.30% and 49.01%, respectively. However, there were sex-specific responses to heavy metals: females suffered more negative effects, as they had significantly greater decreases in root biomass, R/S ratio, height and total chl, and significantly smaller increases in NPT and POD activities than males. The present meta-analysis shows the responses of *Populus* females and males to heavy metals on a regional scale, which is crucial for understanding the patterns of sexual dimorphism and sex ratio biases in *Populus* with increasing heavy metal pollution in the future.

## Introduction

Industrialization and urbanization have increased the emission of heavy metals, which has become a global problem due to the adverse effects on biosystems and public health^[1−4]^. Heavy metals, e.g. cadmium (Cd), lead (Pb), chromium (Cr), copper (Cu), manganese (Mn) and zinc (Zn), are major sources of soil pollution and they receive increasing attention^[5−8]^. It has been widely reported that heavy metal stress negatively affects plant growth, decreases photosynthesis and disturbs biochemical and physiological processes^[4,9,10]^. Heavy metal stress usually increases the production of ROS, such as ${\text{O}^-_2} $ and H_2_O_2_, resulting in oxidative stress. Such increasing levels of ROS lead to lipid peroxidation and the damage of cell structure and membranes^[11−14]^.

Plants act as bioaccumulators that extract and concentrate heavy metals from the soil and water^[13,15,16]^, and they have a series of defense mechanisms to cope with heavy metals. Plants commonly allocate heavy metals into the roots and stems, restrict transportation to the leaves and protect the photosynthetic cells from heavy metal damage^[7,13,17,18]^. In addition, plants can modify gene expression^[19]^, upregulate antioxidant enzymes, such as APX, CAT and POD, scavenge ROS and alleviate the oxidative damage induced by heavy metals^[14,20]^. Many studies have previously investigated plants' responses to heavy metals; however, the knowledge is limited concerning quantitative assessment at regional and global levels, especially in dioecious plants.

Renner^[21]^ reported that there are 15,600 dioecious angiosperm species that account for 5%−6% of all plant species. Under unfavorable conditions, the greater reproduction costs of females to produce flowers, seeds and fruits result in higher sensitivity and worse performance compared to males^[22−25]^. In addition, the different responses in females and males may lead to sex ratio biases that potentially affects the structure and stability of ecosystems^[23,24,26]^. Thus, especially dioecious plants may be at risk and vulnerable to environmental changes due to the sex-specific responses in growth, physiology and morphology under stress conditions, which further reinforces the spatial sexual segregation^[27−29]^.

*Populus* species generally have fast growth rates and they are distributed in the Northern Hemisphere. The small genome size, clonal propagation and commercial values have made *Populus* species excellent model plants to study trees' responses to environmental stresses^[29−33]^. In addition, *Populus* species are dioecious, and separate female and male individuals may have different responses under unfavorable conditions^[34−37]^. For example, *P. cathayana* males have higher plasticity in photosynthetic activity, and females show more severe damage to cellular ultrastructure under Pb stress^[38]^. *P. deltoides* females suffer greater negative effects under Cd stress and show higher levels of leaf symptoms, lipid peroxidation and damage to the cellular ultrastructure^[39]^. Despite some previous research activity, there is still limited quantitative information on the region patterns of the responses of dioecious *Populus* trees to heavy metals.

In the present study, we performed a meta-analysis with 946 sets of comparative data from 20 publications to evaluate the responses of biomass accumulation and allocation, photosynthetic capacity, oxidative stress and antioxidants in *Populus* females and males under heavy metal exposure, such as Cu, Mn, Zn, Pb and Cd. We aimed to answer the following questions: (1) Whether *Populus* females and males also exhibit different responses to heavy metals on a regional scale? If yes, (2) whether *Populus* males perform better and have higher resistance compared to females under heavy metal exposure?

## Materials and methods

### Data collection

Peer-reviewed articles, other academic papers, and book chapters reporting the effects of heavy metals on antioxidant enzyme activities and on the concentration of heavy metals in different organs of male and female poplars published before May 2023 were searched in *Web of Science* and *China National Knowledge Infrastructure* (*CNKI*). We used the following keywords ('sexual' OR 'male and female' OR 'sex-related') AND ('heavy metal' OR 'Cd stress' OR 'Zn stress' OR 'Mn stress' OR 'Pb stress' OR 'Cu stress' OR 'aluminum' OR 'uranium') AND ('enzymatic activity' OR 'reactive oxygen species' OR 'ROS' OR 'MDA' OR 'CAT' OR 'POD' OR 'SOD' OR 'NPT' OR '${\text{O}^-_2} $' OR 'H_2_O_2'_ ) AND ('poplars' OR '*Populus'*) AND ('biomass' OR 'Height' OR 'chlorophyll content' OR 'photosynthetic activity' OR '*g*_s'_ OR '*E'*). We applied the following criteria to select the primary studies: (1) The experiments were controlled experiments, which also had a control treatment; (2) At least antioxidant enzyme activities or ROS or growth or photosynthetic capacity were reported in the included papers; (3) Each paper reported at least one type of a heavy metal treatment; (4) Parameters detected in fewer than six data units were eliminated from the analysis to retain the variability in each observation; (5) The average values and sample sizes of variables, such as growth characters, photosynthetic capacity, and heavy metal content in different organs of poplar trees are directly reported or can be calculated.

The data are mainly extracted from the main text and tables of the primary studies, The GetData Graph Digitizer (version 2.26, www.getdata-graph-digitizer.com) was used to extract data from figures. After extraction and compilation, we had collected a total of 946 sets of comparative data (32 for MDA, 36 for ${\text{O}^-_2}  $, 26 for H_2_O_2_, 16 for TBARS, 52 for SOD, 24 for CAT, 52 for POD, 22 for NPT, 40 for APX, 24 for proline, 18, 22, 10, six and 10 for leaf Cd, Pb, Zn, Mn and Cu, respectively, 10, 22, 10, six and six for stem Cd, Pb, Zn, Mn and Cu, respectively, 12, 22, 10, six and six for root Cd, Pb, Zn, Mn and Cu, respectively, 42 for total biomass, 60 for leaf biomass, 58 for stem biomass, 60 for root biomass, 20 for height, 30 for R/S ratio, 24 for total chl, 54 for *P*_n_, 40 for gs, 44 for *E*, and 14 for *C*_i_) from 20 publications. These were included in our database (Supplemental Table S1). The data distribution for all variables was normal (Supplemental Figs S1, S2, S3). We consider that our data set was suitable for the present analysis.

### Data analysis

We calculated the effect of the heavy metal stress on the growth, photosynthesis capacity, antioxidant capacity, and heavy metal content in different organs of poplar trees. Natural log response ratios (lnRR) for each pairwise comparison were derived using the following equation^[40−42]^:

1 $ {\mathrm{LnRR}} = \left( {\frac{{\overline X t}}{{\overline X {\text{c}}}}} \right) $

Where $\overline  X_t $ and $\overline X_{\rm c} $ are the means of the growth, photosynthesis capacity, antioxidant capacity, and heavy metal content in different organs in heavy metal treatment and control groups, respectively. Because numerous primary studies in our database failed to report standard deviations or standard errors, we used the number of replicates associated with each lnRR as a weight^[43−44]^. The used formula was then as follows:

2 $ Wr=(Nc\times Nt)/(Nc+Nt) $

where *Wr* is the weight associated with each lnRR, and *Nt* and *Nc* are the number of repeats in heavy metal and control groups, respectively.

We calculated the weighted mean effect sizes (lnRR_++_) for the growth, photosynthesis, antioxidant capacity, and heavy metal concentration in different organs. We employed linear mixed-effects models exclusively focusing on the intercept. The response variable for these models was represented by lnRR. Furthermore, we incorporated the identity of primary studies from which the data were collected as a random-effects factor. This factor enabled us to address any potential non-independence of observations derived from the same primary study^[44,45]^. The implementation of the linear mixed effect model and meta-regression was conducted using the restricted Maximum Likelihood Estimation (MLE) method within the lme4 software package^[46]^. To aid the interpretation of results, we back-transformed lnRR_++_ and the associated 95% confidence intervals (CI) using the equation of (e^lnRR++^-1) × 100%^[43]^. All relevant statistical analyses were performed in R version 4.3.1^[47]^. All figures were prepared using Origin 9.0 (OriginLab) software.

## Results

### Heavy metal effects across sexes

**Figure 1:**
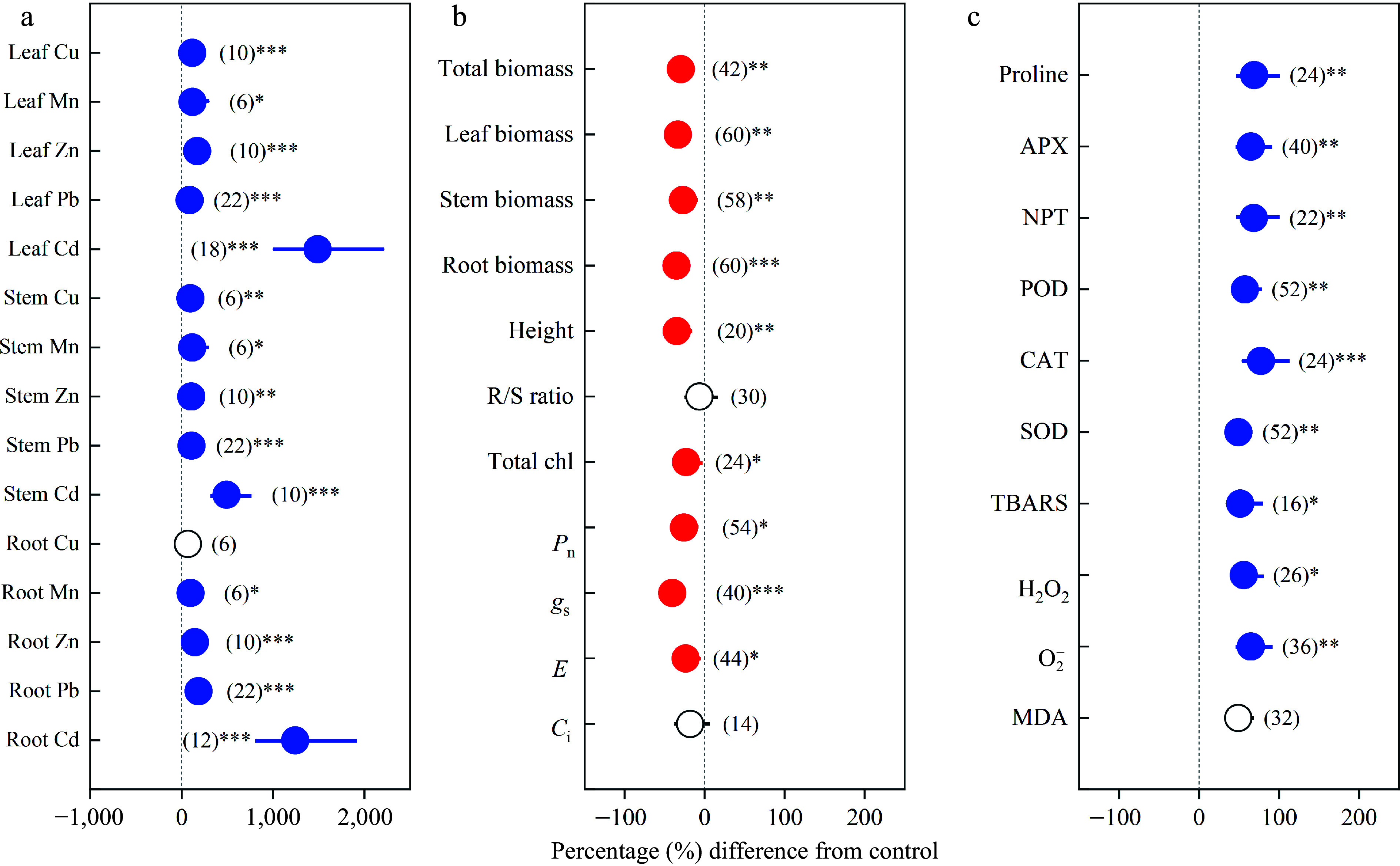
Overall effects of heavy metals on the concentrations of (a) Cu, Mn, Zn, Pb, Cd in leaf, stem and root; (b) total biomass, leaf biomass, stem biomass, root biomass, height, R/S ratio, total chl, *P*_n_, *g*_s_, *E* and *C*_i_; (c) proline, APX, NPT, POD, CAT, SOD, TBARS, H_2_O_2_, ${\text{O}^-_2}  $ and MDA. Values are means with 95% confidence intervals. The number of observations for each variable is shown in parentheses. The blue color indicates significant positive effects, and the red color indicates significant negative effects.^*^p < 0.05,^**^p < 0.01,^***^ *p* < 0.001.

Heavy metals were found to have positive effects on the concentrations of Cu, Mn, Zn, Pb and Cd as well as on oxidative stress and antioxidant enzyme activities, but negative effects on growth and photosynthetic capacity (Fig. 1). The concentrations of heavy metals significantly increased in different organs, except the Cu concentration of roots (Fig. 1a). Under heavy metal exposure, the total biomass, leaf biomass, stem biomass, root biomass, height and R/S ratio decreased by 29.78%, 33.41%, 27.22%, 35.30%, 34.83% and 6.50%, respectively. Furthermore, total chl, *P*_n_, *g*_s_, *E* and *C*_i_ decreased by 23.30%, 26.03%, 40.49%, 23.76% and 18.24%, respectively (Fig. 1b). Oxidative stress variables TBARS, H_2_O_2_, ${\text{O}^-_2} $ and MDA increased in leaves by 51.39%, 55.79%, 64.67% and 48.92%, respectively. The antioxidant enzyme activities, including proline, APX, NPT, POD, CAT and SOD, increased in leaves by 68.91%, 64.81%, 68.40%, 57.34%, 77.30% and 49.01%, respectively (Fig. 1c).

### Sexual differences in heavy metal concentrations, growth and antioxidants

**Figure 2:**
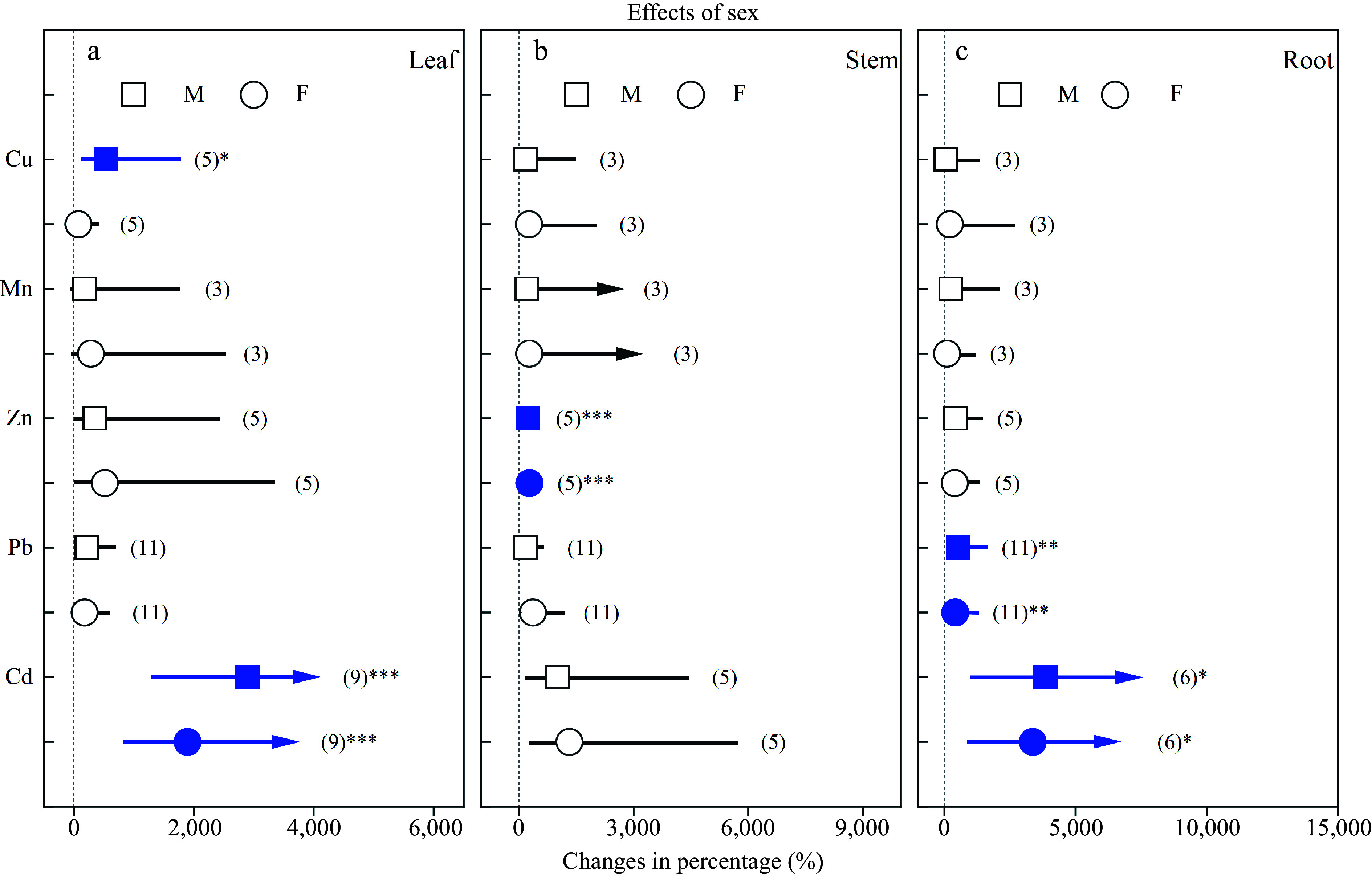
Impacts of sex on the effects of heavy metals on the concentrations of Cu, Mn, Zn, Pb and Cd in (a) leaf, (b) stem and (c) root. Values are means with 95% confidence intervals. The blue color indicates significant positive effects.^*^p < 0.05,^**^p < 0.01,^***^ *p* < 0.001.

Heavy metals significantly increased leaf Cu concentration in males, leaf Cd concentrations in both sexes, stem Zn concentrations in both sexes, and root Pb and Cd concentrations in both sexes (Fig. 2). There were no significant differences in the concentrations of heavy metals between females and males.

**Figure 3:**
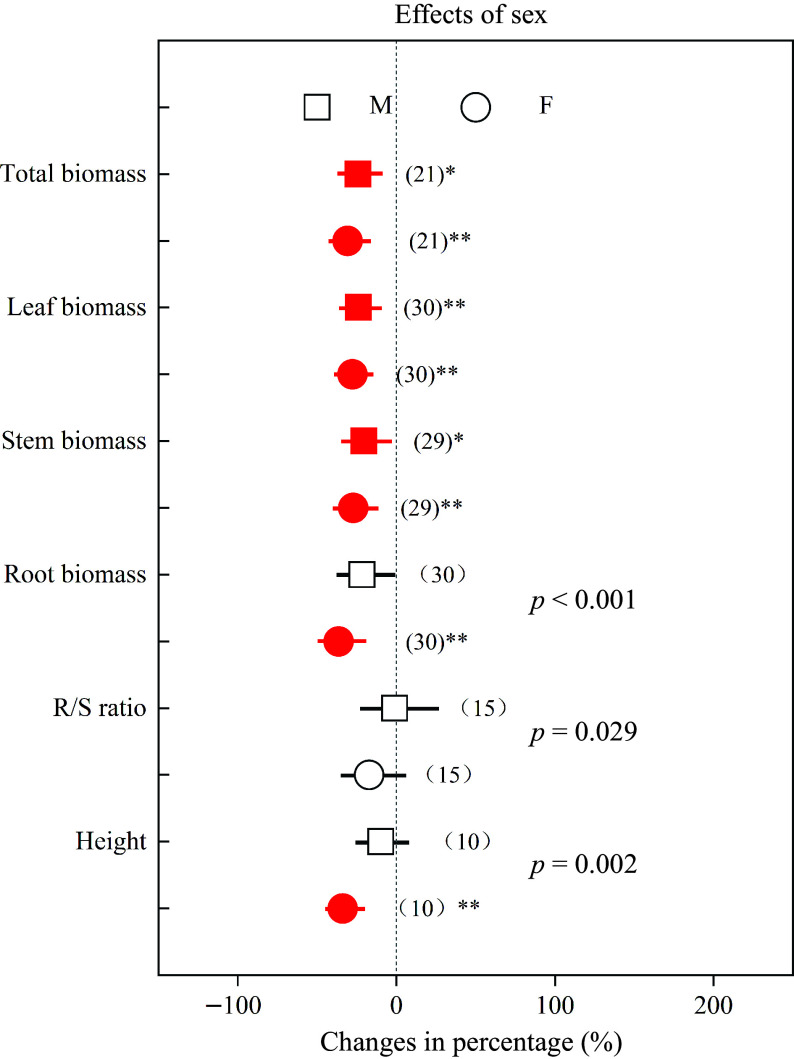
Impacts of sex on the effects of heavy metals on total biomass, leaf biomass, stem biomass, root biomass, R/S ratio and height. Values are means with 95% confidence intervals. The *p*-values indicate differences between *Populus* sexes, and the red color indicates significant negative effects.^*^p < 0.05,^**^p < 0.01,^***^ *p* < 0.001.

**Figure 4:**
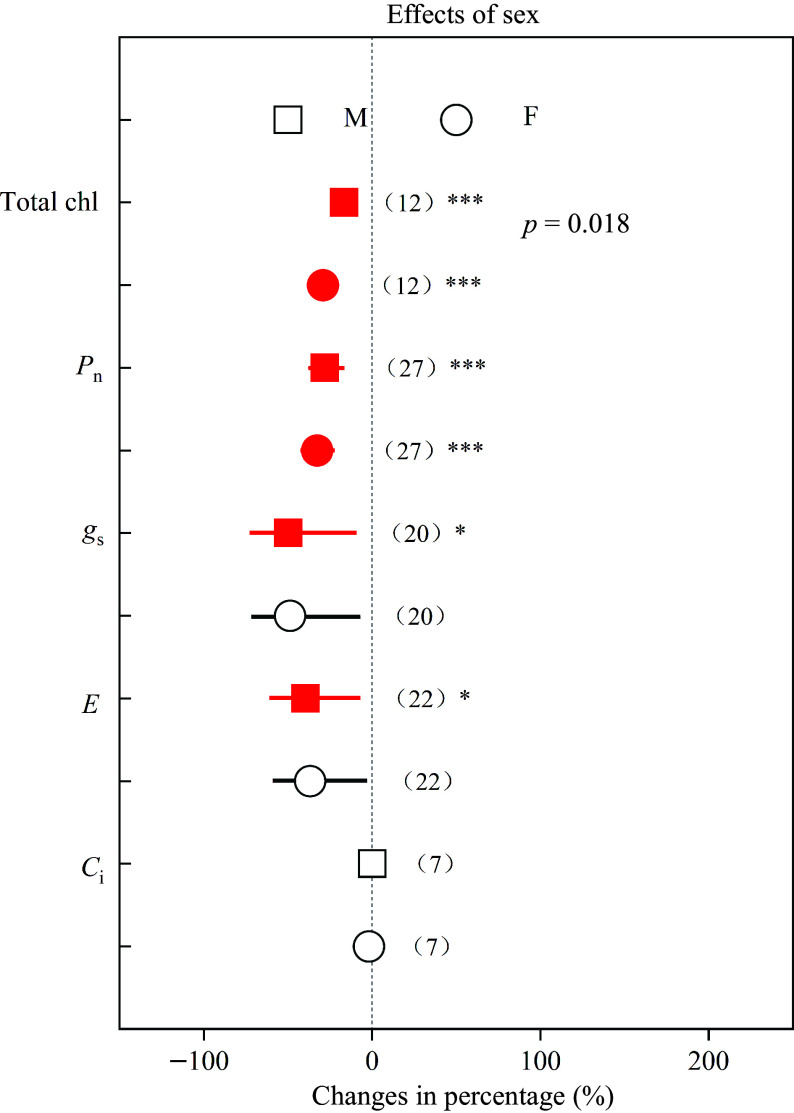
Impacts of sex on the effects of heavy metals on total chl, *P*_n_, *g*_s_ , *E* and *C*_i_. Values are means with 95% confidence intervals. The *p*-values indicate differences between *Populus* sexes, and the red color indicates significant negative effects.^*^p < 0.05,^**^p < 0.01,^***^ *p* < 0.001.

Root biomass, R/S ratio and height decreased in females by 36.43%, 17.15% and 33.87%, respectively, while these parameters decreased in males by 21.63%, 1.11% and 9.85%, respectively (Fig. 3). In addition, total chl, *P*_n_, *g*_s_ and *E* in females decreased by 29.21%, 32.71%, 48.77% and 36.80%, respectively, while in males these parameters decreased by 16.63%, 28.00%, 47.79% and 39.52%, respectively (Fig. 4). Compared with males, *Populus* females showed significantly greater declines (*p* < 0.05) in root biomass, R/S ratio, height and total chl under heavy metal exposure.

**Figure 5:**
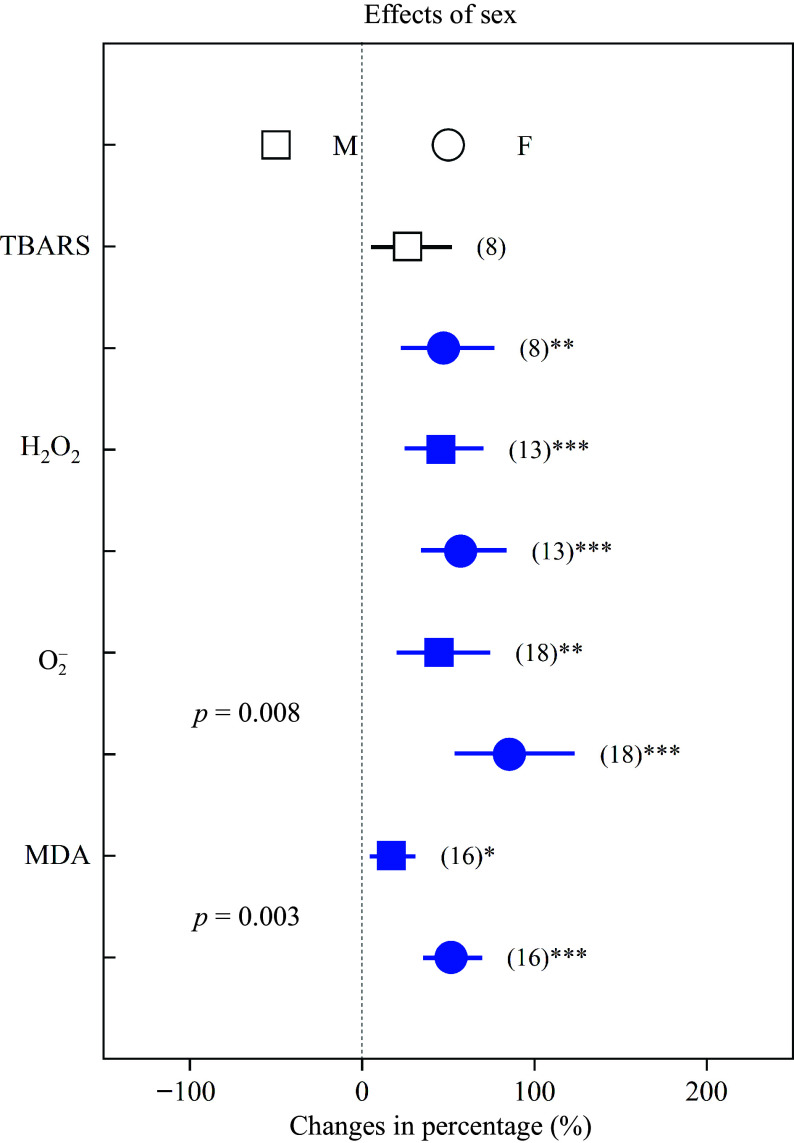
Impacts of sex on the effects of heavy metals on the concentrations of TBARS, H_2_O_2_, ${\text{O}^-_2}  $, and MDA. Values are means with 95% confidence intervals. The *p*-values indicate differences between *Populus* sexes, and the blue color indicates significant positive effects.^*^p < 0.05,^**^p < 0.01,^***^ *p* < 0.001.

**Figure 6:**
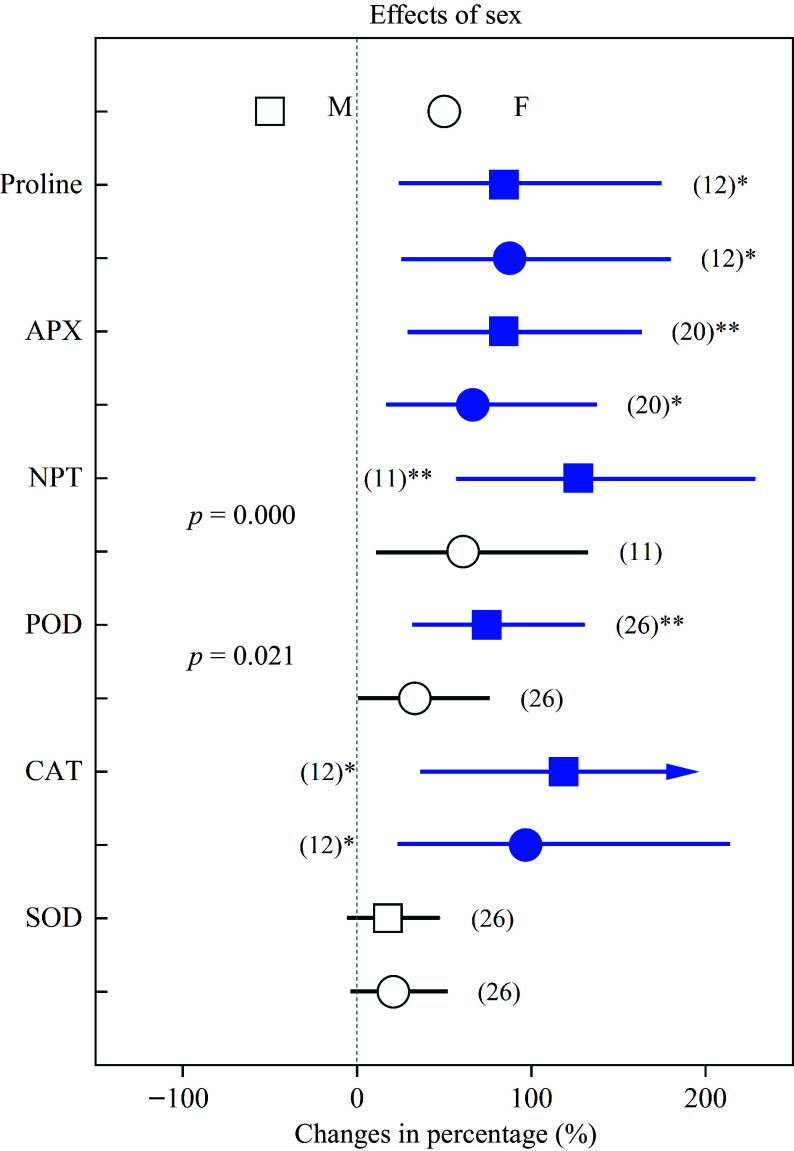
Impacts of sex on the effects of heavy metals on the concentrations of proline, APX, NPT, POD, CAT and SOD. Values are means with 95% confidence intervals. The *p*-values indicate differences between *Populus* sexes, and the blue color indicates significant positive effects.^*^p < 0.05,^**^p < 0.01,^***^ *p* < 0.001.

Heavy metals significantly increased oxidative stress in both sexes, except TBARS in males. ${\text{O}^-_2}  $ and MDA of females increased by 85.41% and 51.54%, respectively, whereas these parameters were significantly lower (*p* < 0.05) in males, in which they increased by 44.70% and 17.09%, respectively (Fig. 5). In addition, NPT and POD increased in females by 60.79% and 33.16%, respectively, while in males they were significantly higher (*p* < 0.05) and increased by 127.12% and 74.53%, respectively (Fig. 6).

## Discussion

### Heavy metal accumulation and allocation

In the present study, heavy metal treatment significantly increased average concentrations of Cu, Mn, Zn, Pb and Cd in different organs, except Cu in roots (Fig. 1a), which agreed with previous reports showing that *Populus* trees, as the bioaccumulators, can extract heavy metals from contaminated soils^[38,48,49]^. In addition, *Populus* can inhibit heavy metal transport to leaves and allocate more into stems and roots^[39,50,51]^. Our results were in line with the above statements. Leaf Cu concentration in males increased significantly more than that in females under Cu exposure. The reason was that the transpiration stream is the main way for Cu transport from roots to shoots^[50,52,53]^. At the same time, the average stem and root Cu concentrations of males increased by 170.28% and 63.20%, respectively, while these of females increased by 258.33% and 207.47%, respectively, implying males having a better ability to inhibit Cu transportation.

We found that Pb concentrations of roots in both sexes significantly increased, while Pb concentrations of leaves and stems showed no significant differences compared with the control treatments. These results indicated that the Pb accumulation was higher in roots, which is consistent with previous studies^[54−56]^. Cd is a highly toxic heavy metal for both plants and humans. A recent study have found that Cd translocation and reallocation was sex-dependent and that females showed greater upward transport of Cd, whereas males had greater downward transport, indicating males had greater capacity to restrict Cd transportation and protect the photosynthetic cells from heavy metal damage^[8]^. Some earlier studies have reported that Cd absorption and accumulation are affected by nitrogen levels^[8,57]^ and plant-plant interactions^[18]^, which indicate the complex nature of heavy metal absorption and accumulation and may explain the non-significant differences in the concentrations of heavy metals between *Populus* females and males shown by the present meta-analysis.

### Growth and photosynthetic capacity affected by heavy metals

Similarly as documented elsewhere^[7,8,20,50,53,55,56]^, we found that heavy metals have negative effects on plant growth, total chl, and photosynthetic capacity in both sexes. On the other hand, root biomass and height in females decreased by 36.43% and 33.87%, respectively, while these parameters decreased in males less, namely by 21.63% and 9.85%, respectively (Fig. 3). Compared with males, females had significantly greater decreases in root biomass, R/S ratio and height, indicating that females may be more sensitive and suffer greater negative effects, which was in accordance with earlier studies^[50,53,55]^. Previous studies have demonstrated that plant roots play key roles in the absorption of nutrients and water^[58−60]^. Females with smaller root biomass and R/S ratio may have a lower capacity to absorb resources, and this could explain the more negative effects observed in females under heavy metal stress. Previous studies have reported that Pb and Cd stress induced more severe damage and decreased number of chloroplasts in females^[38, 53]^, which may explain that females had a significantly greater decline in total chl (Fig. 4), and implying that *Populus* males have more efficient heavy metal responses. Thus, the photosynthetic pigments of males may be better protected, while the photosynthesis machinery of females is more sensitive to heavy metal stress^[50]^.

### Oxidative stress and antioxidants affected by heavy metals

It is well known that heavy metals can result in an enhanced ROS accumulation, and they can cause oxidative damage to cellular membranes and proteins^[21,35,61]^. We discovered in the present study that heavy metals induce oxidative stress, for instance, the levels of TBARS, H_2_O_2_, ${\text{O}^-_2}  $ and MDA increased by approximately 50%. Furthermore, ${\text{O}^-_2}  $ and MDA in females increased by 85.41% and 51.54%, respectively, whereas these parameters increased significantly less in males, namely by 44.70% and 17.09%, respectively (Fig. 5). Previous studies have reported that MDA usually indicates lipid peroxidation levels in plants and ${\text{O}^-_2}  $ is used to assess the levels of oxidative damage under stress conditions^[37,60,62,63]^. The results of the present analysis indicated that heavy metals induce more serious oxidative damage on *Populus* females, which agreed with previous reports that males are more tolerant to heavy metals^[50,53−56]^.

On the other hand, plants usually upregulate their antioxidant enzyme activities to cope with heavy metals. We found that the levels of APX, NPT, POD, CAT and SOD increased by 64.81%, 68.40%, 57.34%, 77.30% and 49.01%, respectively, which closely correlated with oxygen-scavenging and indicated important roles for antioxidant enzymes when dealing with abiotic stress^[32,60,62,63]^. In addition, NPT and POD increased significantly more in males than females. The above statements collectively demonstrated that *Populus* males have higher antioxidant enzyme activities, better abilities to scavenge ROS and a more effective antioxidant defense system under heavy metals^[7,8,50,51]^.

Previous studies have demonstrated that sexual dimorphism in *Populus* may lead to niche segregation, bias in sex ratios, and spatial segregation of the sexes (SSS) across different environmental gradients^[22,28,29]^. Less stress and resource-rich areas are usually with female-biased sex ratios, whereas males are more abundant under adverse and stressful conditions^[22]^. Thus, dioecious plants are more vulnerable under the future climate change due to SSS across environmental gradients^[27]^. The sex-specific responses and adaptive strategies of *Populus* may result in a situation that one sex is more prone to future climate change than the other one. These results are important for understanding sexual dimorphism, spatial sexual segregation and sex ratio biases, which may be reinforced in *Populus* with the increasing heavy metal pollution in the future.

## Conclusions and perspectives

In the present meta-analysis, we quantified the responses of growth, photosynthetic capacity, oxidative stress and antioxidants in *Populus* females and males to heavy metals at a region scale. Heavy metals have negative effects on *Populus* growth and photosynthetic capacity, and increased oxidative stress and antioxidants. Although we did not discover significant differences in the concentrations of heavy metals between females and males, there were still some sex-specific responses to heavy metals. Females suffered more negative effects, as they showed significantly more increased ${\text{O}^-_2} $ and MDA levels, significantly less increased NPT and POD activities, a significantly more decreased root biomass and R/S ratio, height and total chl. Our study is the first to present how *Populus* females and males respond to heavy metals on a regional scale. In addition, it is needed to investigate sexual responses to heavy metals in the field. Further studies are essential to explore the adopted strategies and mechanisms of *Populus* females and males to cope with different heavy metal stress. Previous studies have reported that selenium and silicon addition can improve plant tolerance, and more research is needed to clarify the mechanism. Multi-omics technologies, i.e., transcritomics, metabolomics, proteomics, etc. as well as more metabolite databases and advanced analytical tools can improve our understanding of sex-related molecular mechanisms in the future.

## SUPPLEMENTARY DATA

Supplementary data to this article can be found online.

## Data Availability

The data that support the findings of this study are available from the corresponding author upon reasonable request.
