# Supplementary material for: Responses of dioecious Populus to heavy metals: a meta-analysis
Source: For Res (Fayettev). 2023 Oct 24;3:25. doi: 10.48130/FR-2023-0025 (PMC11524290; doi:10.48130/FR-2023-0025)
Supplement: Supplementary file 1 — Supplementary data to this article can be found online. [file FR-2023-0025-S1.zip › 10.48130_FR-2023-0025-Suppl-FigureS2.pdf]

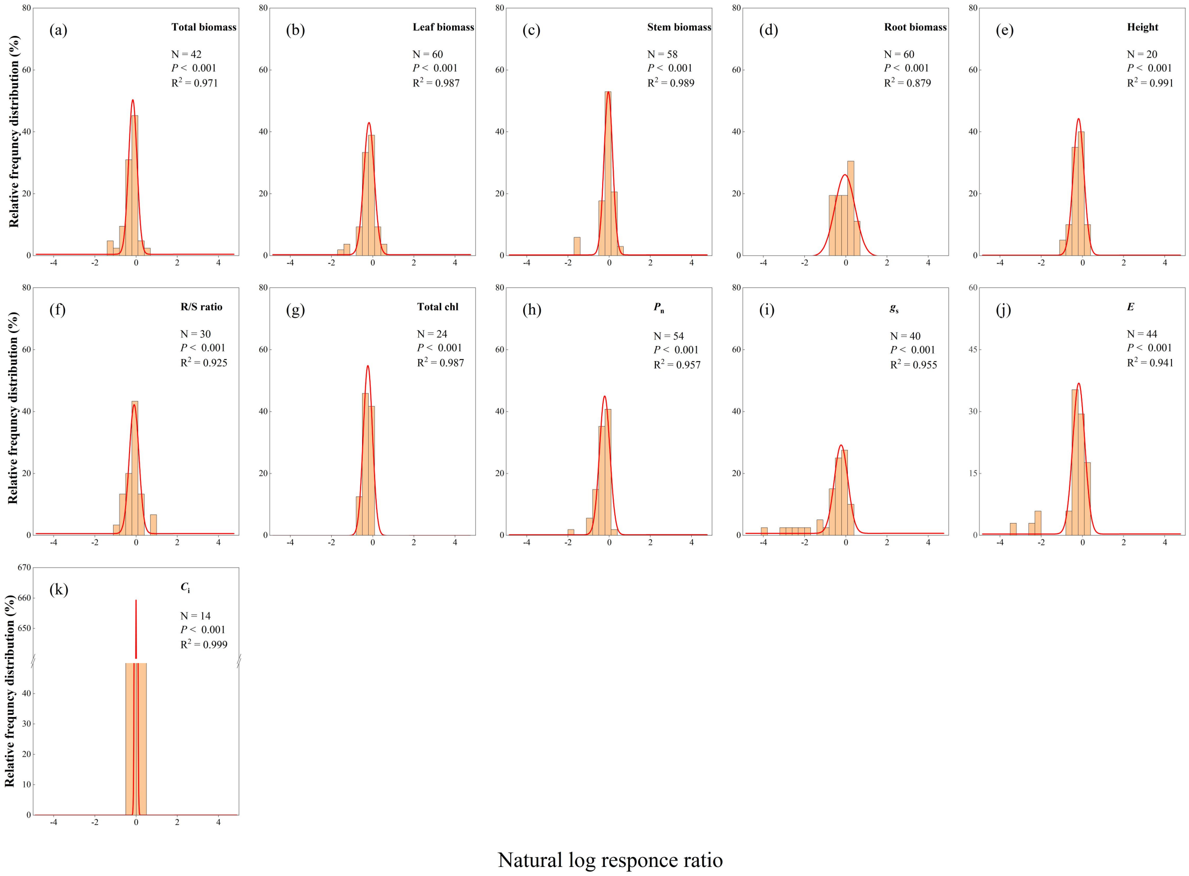

Figure S2 Frequency distribution of the data for heavy metal concentrations of (a) Total biomass, (b) Leaf biomass, (c) Stem biomass, (d) Root biomass, (e) Height (f) R/S ratio, (g) Total chl, (h) P_n, (i) g_s, (j) E, and (k) C_i
