# Supplementary material for: Responses of dioecious Populus to heavy metals: a meta-analysis
Source: For Res (Fayettev). 2023 Oct 24;3:25. doi: 10.48130/FR-2023-0025 (PMC11524290; doi:10.48130/FR-2023-0025)
Supplement: Supplementary file 1 — Supplementary data to this article can be found online. [file FR-2023-0025-S1.zip › 10.48130_FR-2023-0025-Suppl-FigureS3.pdf]

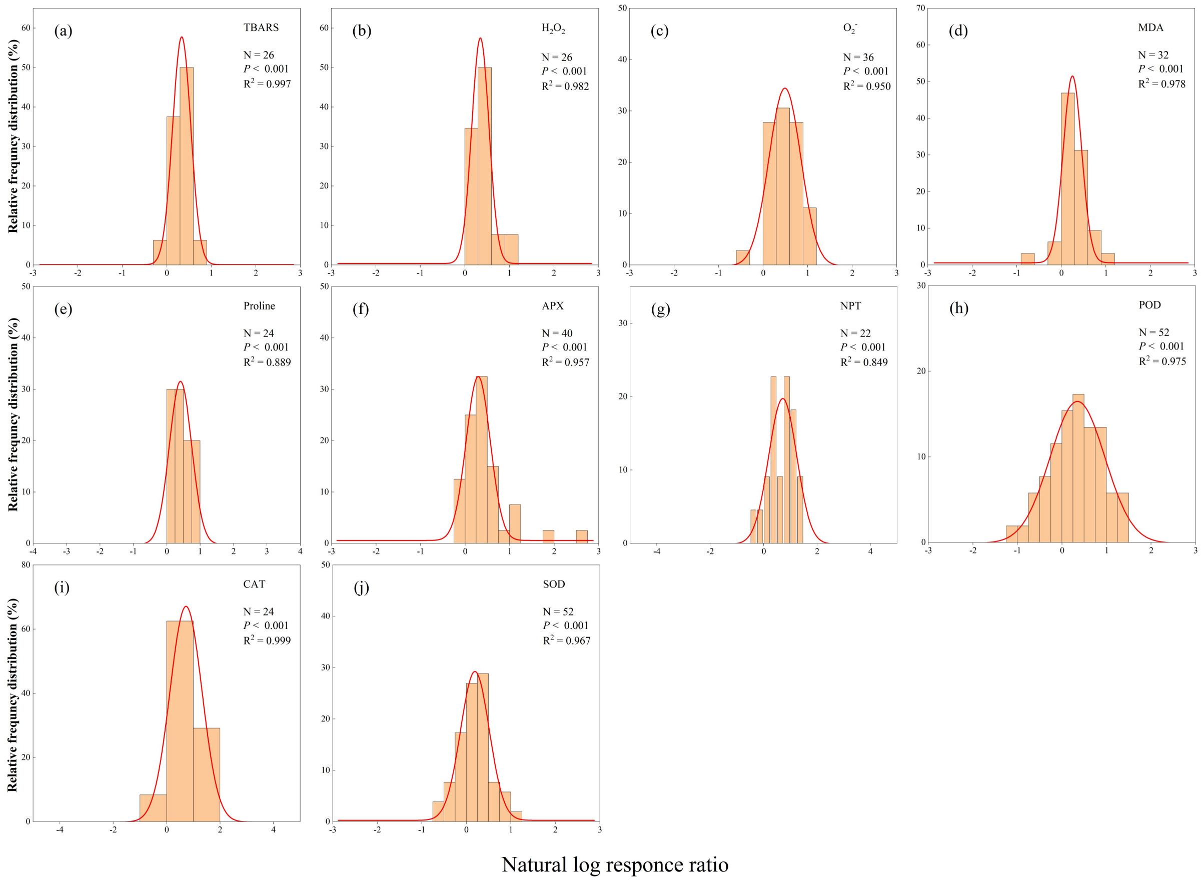

Figure S3 Frequency distribution of the data for heavy metal concentrations of (a) TBARS, (b) H_2O_2, (c) O_2^-, (d) MDA, (e) Proline, (f) APX, (g) NPT, (h) POD, (i) CAT, and (j) SOD.
