# Supplementary material for: Responses of dioecious Populus to heavy metals: a meta-analysis
Source: For Res (Fayettev). 2023 Oct 24;3:25. doi: 10.48130/FR-2023-0025 (PMC11524290; doi:10.48130/FR-2023-0025)
Supplement: Supplementary file 1 — Supplementary data to this article can be found online. [file FR-2023-0025-S1.zip › 10.48130_FR-2023-0025-Suppl-Tables1.pdf]

Table S1 A summary of the sample sizes, and ranges of publication year and effect size (lnRR) for each soil variable.

Parameter	Sample size	Range of publication year	Range of lnRR
Leaf Cd	18	2011－2023	1.697－4.600
Leaf Pb	22	2013－2021	-0.573－3.908
Leaf Zn	10	2013－2021	0.589－3.189
Leaf Mn	6	2018	-0.047－2.389
Leaf Cu	10	2013－2020	-0.534－3.409
Stem Cd	10	2016－2019	1.336－3.579
Stem Pb	22	2013－2021	0.060－4.536
Stem Zn	10	2013－2021	0.877－1.703
Stem Mn	6	2018	-0.941－3.108
Stem Cu	6	2020	-0.375－2.371
Root Cd	12	2016－2022	2.057－5.298
Root Pb	22	2013－2021	-2.197－5.191
Root Zn	10	2013－2021	0.621－2.571
Root Mn	6	2018	-0.504－2.222
Root Cu	6	2020	-1.099－2.285
Total biomass	42	2011－2021	-1.359－0.483
Leaf biomass	60	2012－2022	-1.438－0.623
Stem biomass	58	2012－2022	-1.622－0.465
Root biomass	60	2012－2022	-1.565－0.511
Height	20	2020－2023	-0.984－0.202
R/S ratio	30	2012－2019	-0.916－0.891
Total chl	24	2012－2023	-0.671－-0.019
P_n	54	2011－2021	-1.712－0.161
g_s	40	2011－2021	-3.979－0.199
E	44	2011－2021	-3.356－0.340
C_i	14	2012－2021	-0.098－0.077
Proline	24	2013－2022	0.062－1.797
APX	40	2011－2022	-0.188－2.552
NPT	22	2011－2020	-0.326－1.310
POD	52	2011－2022	-1.058－1.342
CAT	24	2013－2021	-0.399－1.695
SOD	52	2011－2022	-0.698－1.147
TBARS	16	2011－2016	-0.175－0.610
H_2O_2	26	2011－2022	0.031－1.158
O_2^-	36	2011－2022	-0.372－1.096
MDA	32	2012－2022	-0.625－1.025
