# Supplementary material for: Responses of dioecious Populus to heavy metals: a meta-analysis
Source: For Res (Fayettev). 2023 Oct 24;3:25. doi: 10.48130/FR-2023-0025 (PMC11524290; doi:10.48130/FR-2023-0025)
Supplement: Supplementary file 1 — Supplementary data to this article can be found online. [file FR-2023-0025-S1.zip › 10.48130_FR-2023-0025-Suppl-FigureS1.pdf]

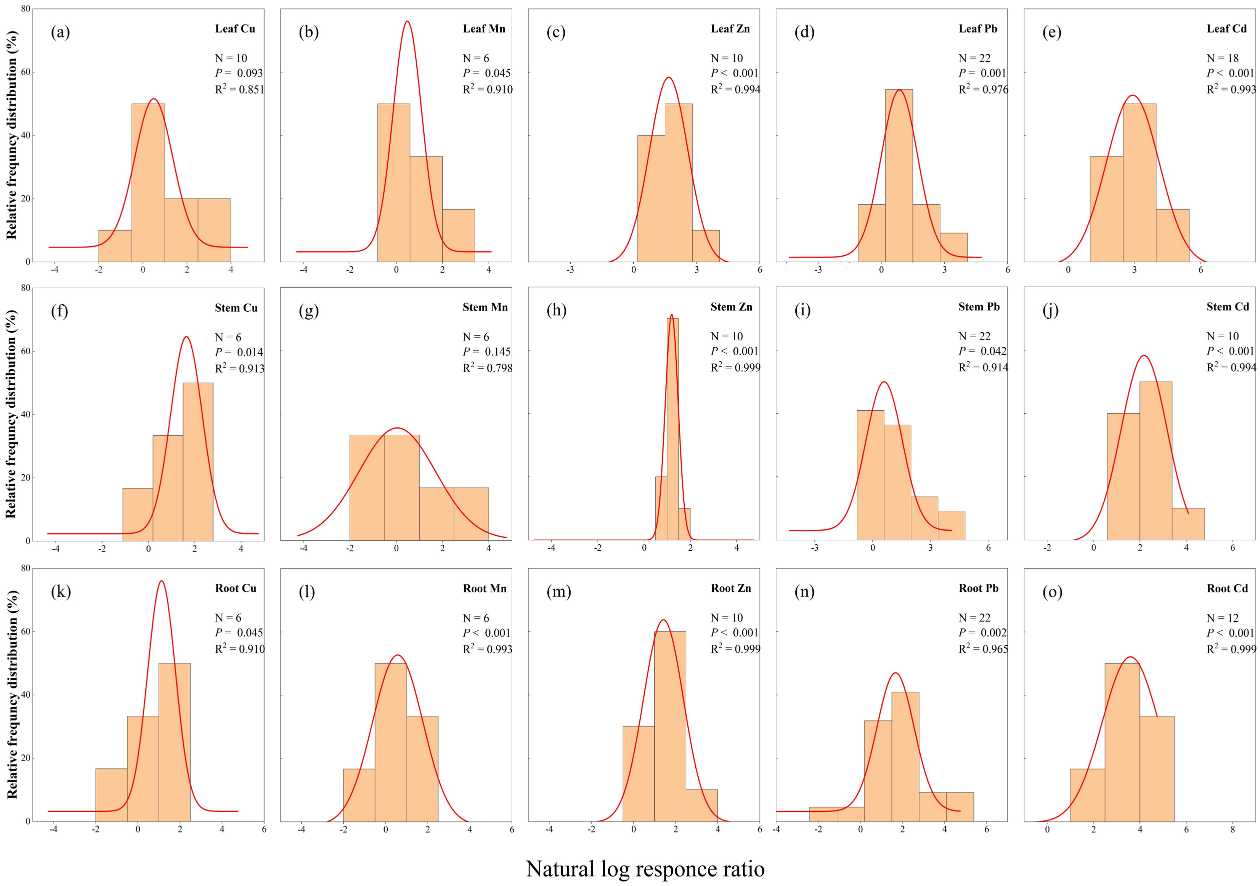

Figure S1 Frequency distribution of the data for heavy metal concentrations of (a) Leaf Cu, (b) Leaf Mn, (c) Leaf Zn, (d) Leaf Pb, (e) Leaf Cd, (f) Stem Cu, (g) Stem Mn, (h) Stem Zn, (i) Stem Pb, (j) Stem Cd, (k) Root Cu, (l) Root Mn, (m) Root Zn, (n) Root Pb, and (o) Root Cd.
